# Osteopathic manipulative treatment and its relationship to autonomic nervous system activity as demonstrated by heart rate variability: a repeated measures study

**DOI:** 10.1186/1750-4732-2-7

**Published:** 2008-06-05

**Authors:** Charles E Henley, Douglas Ivins, Miriam Mills, Frances K Wen, Bruce A Benjamin

**Affiliations:** 1 University of Oklahoma Health Sciences Center, Department of Family Medicine, Tulsa, OK 74120, USA; 2 Oklahoma State University Center for Health Sciences Department of Osteopathic Manipulative Medicine, Tulsa, OK 74107, USA; 3 Oklahoma State University Center for Health Sciences Department of Pharmacology and Physiology, Tulsa, OK 74107, USA

## Abstract

**Background:**

The relationship between osteopathic manipulative treatment (OMT) and the autonomic nervous system has long been acknowledged, but is poorly understood. In an effort to define this relationship, cervical myofascial release was used as the OMT technique with heart rate variability (HRV) as a surrogate for autonomic activity. This study quantifies that relationship and demonstrates a cause and effect.

**Methods:**

Seventeen healthy subjects, nine males and eight females aged 19–50 years from the faculty, staff, and students at Oklahoma State University Center for Health Sciences College of Osteopathic Medicine, acted as their own controls and received interventions, administered in separate sessions at least 24 hours apart, of cervical myofascial OMT, touch-only sham OMT, and no-touch control while at a 50-degree head-up tilt. Each group was dichotomized into extremes of autonomic activity using a tilt table. Comparisons were made between measurements taken at tilt and those taken at pre- and post-intervention in the horizontal.

The variance of the spectral components of HRV, expressed as frequencies, measured the response to change in position of the subjects. Normalized low frequency (LF) and high frequency (HF) values, including LF/HF ratio, were calculated and used to determine the effect of position change on HRV.

**Results:**

Predominantly parasympathetic responses were observed with subjects in the horizontal position, while a 50-degree tilt provided a significantly different measure of maximum sympathetic tone (p < 0.001). Heart rate changed in all subjects with change in position; respirations remained constant. When OMT was performed in a sympathetic environment (tilt), a vagal response was produced that was strong enough to overcome the sympathetic tone. There was no HRV difference between sham and control in either the horizontal or tilt positions.

**Conclusion:**

The vagal response produced by the myofascial release procedure in the maximally stimulated sympathetic environment could only have come from the application of the OMT. This demonstrates the association between OMT and the autonomic nervous system. The lack of significance between control and sham in all positions indicates that HRV may be a useful method of developing sham controls in future studies of OMT.

**Trial registration:**

clinicaltrials.gov NCT00516984.

## Background

For most osteopathic physicians the validation of osteopathic manipulative treatment (OMT) has been largely observational and based on patient outcomes such as improvement in pain scales, range of motion, and other empiric measures.[1,2] However, the osteopathic profession has long recognized a relationship between the autonomic nervous system and the function of the body in health and disease, although there is relatively little quantitative data evaluating the relationship between manipulation and the autonomic nervous system.[3,4]

A theoretical basis for the action of OMT and its effect in the body has been advanced based on autonomic activation causing concomitant vasodilatation, smooth muscle relaxation, and increased blood flow, resulting in improved range of motion, decrease in pain perception, and/or change in tissue. Until recently this association remained largely a theoretical consideration due to the inability to accurately measure autonomic activity directly. Over the past two decades indirect methods have been developed and refined to provide noninvasive markers of autonomic balance,[5,6] with heart rate variability (HRV) being commonly used. HRV is based on the inherent variation of the R to R intervals of a standard electrocardiogram (ECG), with these variations largely due to changes in autonomic balance at the sinus node. [6-8]

Spectral analysis of heart rate variability has been used to study autonomic balance in humans, and it is generally accepted that the high frequency (HF) component (0.15–0.4 Hz) can be used as a marker for vagal modulation of heart rate. Although it is tempting to use the low frequency (LF) component (0.04–0.15 Hz) as a marker for sympathetic activity, its specificity is less clear. Pagani and colleagues[9,10] have hypothesized that when the LF component is expressed in normalized units (LF_nu_) it becomes a better marker of sympathetic modulation of heart rate. For most studies using spectral analysis, the LF/HF ratio is used and considered by many to be a good index of sympathovagal balance.[6,7,9,10]

The confidence given to the LF/HF ratio accurately reflecting autonomic balance is significantly influenced by experimental design. A tilt protocol involving postural change from horizontal to upright can be used to calibrate the change in the LF/HF ratio which occurs between the two positions and thus set a physiological range for sympathetic and vagal modulation of heart rate. An experimental procedure can then be implemented where comparisons are made of the changes in the LF/HF ratios that occur when the body is shifted from the horizontal to the upright position under conditions with application of an intervention versus without the intervention. In this manner, an experimentally mediated change in LF/HF ratio (i.e., with intervention) can be calibrated against a physiologically relevant change in ratio (i.e., without intervention).

This approach was used by our group in a pilot study (unpublished observations; n = 9 healthy, adult volunteers, 3 females and 6 males) which showed that the LF/HF ratio changed from a mean of 1.75 ± 1.40 (mean ± SD) in the horizontal position to a mean of 6.00 ± 1.20 in the 50-degree head-up position. This change reflects an increase in sympathetic tone. Mean heart rate in these subjects increased from 61 ± 7 bpm to 78 ± 2 bpm in the head-up position. The subjects were then treated in the 50-degree head-up position with an OMT procedure, cervical myofascial release, which is thought to increase vagal tone. After the procedure was applied, the LF/HF ratio decreased back down to 1.75 ± 1.58, even though the subjects were still in the head-up position. These data support the initial hypothesis that specific OMT procedures can modulate vagal tone, and also provide information relating to the significance of the LF/HF change. That is, the application of OMT reversed the increase in the ratio that occurs in the 50-degree head-up position.

We conducted a continuation project to further examine the association between OMT and autonomic nervous system activity as demonstrated by HRV, studying the hypothesis that cervical myofascial release increases vagal tone. In a within subjects (repeated measures) design, we examined the effect of OMT on HRV in comparison with sham treatment (touch only) and control (no touch) conditions.

## Methods

### Recruitment

Thirty (30) persons from the undergraduate medical school class, staff, and faculty at the Oklahoma State University College of Osteopathic Medicine, Tulsa, Oklahoma, were interviewed for the study after answering a standard advertisement soliciting healthy subjects. Twenty-eight (28) study subjects then were selected by their response to a general questionnaire, which indicated suitability for the study, and assessed by the following inclusion criteria: written informed consent, normal healthy adults older than 19 years and younger than 50 years, normal ECG, and normal blood pressure based on criteria published in the Seventh Report of the U.S. Joint National Committee on Prevention, Detection, Evaluation, and Treatment of High Blood Pressure (JNC-7).[11]. Exclusion criteria included chronic cardiovascular disease (heart failure, myocardial infarction, or hypertension), diabetes, asthma, pregnancy, smoking, premature ventricular contractions exceeding 20% of total heart beats, resting supine heart rate greater than 75 bpm or less than 45 bpm, systolic blood pressure greater than 140 mmHg or less than 90 mmHg, or failure of heart rate to increase with passive tilt (50-degrees head-up). Long-distance runners and other conditioned athletes also were excluded.

The study proposal was submitted and approved after full review of both the Oklahoma State University College of Osteopathic Medicine (IRB#2000417) and the University Of Oklahoma College Of Medicine (IRB#12024) Institutional Review Boards.

### General study design

**Figure 1:**
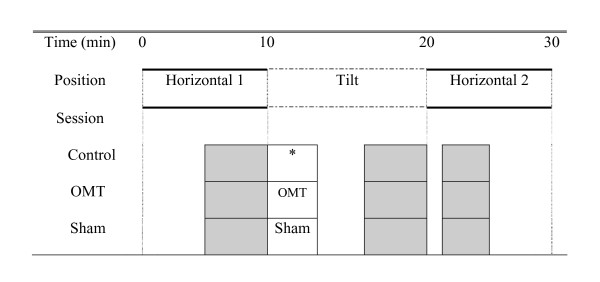
**Experimental protocol**. Shaded areas show the times when data were analyzed for each subject. *= No treatment

The general experimental design is shown in Figure 1. There were three experimental conditions: a) control involving no intervention; b) OMT treatment involving cervical myofascial release, and c) sham treatment involving the placement of hands in the cervical region. The protocol lasted 30 minutes. Changes in body position (horizontal vs 50-degree head-up tilt) were incorporated into the study to facilitate interpretation of autonomic tone measures. ECG data were recorded continuously throughout the experiment. Analysis of autonomic tone was standardized and occurred in the last 5 minutes of horizontal 1 period, the last 5 minutes of tilt, and during the first 5 minutes of heart rate recovery in horizontal 2 period, as shown by the shaded sections of Figure 1. Treatment, either OMT or sham, was administered for 2 minutes and started 30 seconds after tilt started.

Subjects who met inclusion criteria were enrolled in the study and consent was obtained. In a repeated measures design, the subjects acted as their own controls and participated in each of three separate sessions during which only one of the experimental conditions (control, OMT, sham) was administered. The order of administering the experimental conditions was partly randomized. Specifically, at the first session, each subject was randomly assigned to one of the two treatment conditions, OMT or sham. Subjects were then administered the other treatment condition during the second session. The control condition was administered as the third condition. The subjects were not told which treatment they were given at the time. Random assignment to treatment conditions was conducted to control for within subject variations that might affect physiological responses. Only data for subjects who completed all three sessions were analyzed and reported here.

After first obtaining a physician-read normal resting ECG, subjects were randomly assigned to a treatment condition to begin the study. Subjects completed all three experimental sessions with a minimum of 24 hours separating each session. Each session was conducted at the same time of day, between 1:30 p.m. and 4:00 p.m. The subjects were instructed to eat a light lunch without caffeine, no later than 12:00 noon. When they entered the exam room, ECG electrodes were attached and the subject placed on the tilt table. During an initial equilibration period the subjects were acclimated to the tilt table by moving them from the horizontal to head-up position several times. This usually took about 5 minutes. The change in body position was completely passive for the subjects. They did not exert any effort or assist in the change of position.

After equilibration, the following protocol was followed for the control session: 10 minutes at horizontal, 10 minutes at 50-degrees head-up tilt, then back to horizontal for 10 minutes. No treatment was given during the control session. For the OMT and sham treatment sessions, the cervical myofascial release or sham procedure was initiated 30 seconds after the subject was placed in the head-up position and continued for two minutes. Preliminary studies (unpublished observations from our pilot study of nine subjects) indicated that it took subjects an average of 30 seconds to reach a new steady state in heart rate after repositioning to head-up. At the end of treatment phase (control, OMT, or sham), the subjects remained in the upright position for approximately 8 more minutes, and then were returned to the horizontal position for a final 10 minute period.

The cervical myofascial release technique used in this study was adopted from the monograph of Common OMT Techniques by Kenneth Graham, Oklahoma State University Center for Health Sciences.[12] The sham procedure was accomplished by placing the researcher's hands in the same position on the neck as would occur if initiating the cervical myofascial release technique. The position is maintained for the same amount of time as the OMT; however no pressure is applied to the cervical area.

### Data collection

A three lead ECG was recorded from the subject and at the same time a respiratory trace was recorded with a piezo chest transducer RM-204 (iWorx; Dover, NH). Data were collected by an analog-to-digital converter (iWorx HK 214; Dover, NH) with a sampling frequency of 200 Hz and stored on a personal computer for later analysis.

Digital records were analyzed after the experiment. R-R intervals were determined by an R-wave peak detection algorithm. Each data record was reviewed manually. Time series analysis consisted of 300 seconds of data containing consecutive intervals of sinus rhythm. Spectral analysis was carried out with HRV analysis software from the Biomedical Signal Analysis Group, Department of Physics, University of Kuopio, Finland. The 300 second R-R intervals were interpolated by cubic-spline and resampled at 2 Hz to obtain equidistant data samples. The power spectrum was computed by Fourier Transform. Total power was defined in the range of 0.01–0.40 Hz. Low frequency power was defined as 0.04–0.15 Hz and high frequency power as 0.15–0.40 Hz. Normalization of LF and HF indices was used to minimise the impact of a change of the total power in the analysis of the balance between LF and HF. Normalization of power in LF and HF ranges were calculated as the ratio of the power in the considered frequency band to the sum of the HF and LF powers: LF_nu _= 100*LF/(HF+LF); HF_nu _= 100*HF/(HF+LF). The ratio of LF_nu_/HF_nu _was used as an index of autonomic balance.

### Statistical analysis

The study is a two-way (3 × 3) repeated measures design, with each subject acting as their own control. Analysis of the data was conducted using within subjects analysis of variance (ANOVA). Factors were three experimental conditions (Control, OMT, Sham) and three positions (Horizontal 1, Tilt, Horizontal 2). Dependent variables were heart rate, normalized LF (LF_nu_), normalized HF (HF_nu_), the ratio of LF_nu_/HF_nu _(LF/HF ratio), and respiration rate. Post-hoc comparisons were performed with Bonferroni adjustment for multiple comparisons. Pilot study data suggested a large difference in frequencies between conditions indicating that approximations of sphericity would be met without using correction factors. All data were analyzed using SPSS version 12. A power analysis, based on the pilot data, showed a need for an N of 14 using an alpha of 0.05 and a beta of 0.20 (1-beta of 0.80), with an estimated 25% difference in mean ratios for the three conditions and a standard deviation of 3.6.

### Data handling and security

Data acquisition and analysis was accomplished on a laptop computer which was never connected to the Internet. It was password protected and stored under lock and key when not in use. All subjects received a data ID number that was used to code their records. The file linking subject name to ID was destroyed at the end of the experiment.

## Results

Seventeen (61%) of 28 subjects completed all three phases of the study and had clean HRV data to analyze. The 17 subjects that completed the study exceeded our estimated need for an N of 14 by three subjects. There were no adverse effects of the study. Analysis of the subjects not completing the study showed that two of the 28 subjects did not have an increase in heart rate in response to head-up tilt. Nine of the 28 had scheduling conflicts or moved out of town before the data collection was completed. The 17 subjects completing the study consisted of 9 men and 8 women. Their average systolic blood pressure was 120 mmHg ± 2 and diastolic blood pressure was 71 mmHg ± 2. Table 1 shows mean and 95% confidence intervals for heart rate, LF_nu_, HF_nu_, and LF/HF parameters for the three conditions at each position.

**Table 1:** Mean and 95% Confidence Intervals (CI) for Heart Rate, LFnu, HFnu, and LF/HF Parameters

		Control			Sham OMT			OMT		
		
			95% CI			95% CI			95% CI	
		
	Position	Mean	Lower Bound	Upper Bound	Mean	Lower Bound	Upper Bound	Mean	Lower Bound	Upper Bound
Heart Rate (bpm)	H1	67.61	61.79	73.44	65.64	60.42	70.86	62.43	57.89	66.96
	Tilt	80.51	74.52	86.51	78.73	72.03	85.43	71.74	66.29	77.20
	H2	65.43	59.85	71.01	64.03	58.74	69.32	59.53	55.44	63.62

LFnu	H1	53.21	44.05	62.36	44.97	35.63	54.31	40.08	28.80	51.35
	Tilt	80.19	76.00	84.38	76.93	71.25	82.61	58.33	49.87	66.79
	H2	53.30	43.90	62.70	47.98	39.32	56.64	41.49	30.48	52.50

HFnu	H1	47.89	38.41	57.38	54.43	44.53	64.33	59.37	47.37	71.37
	Tilt	20.15	15.73	24.57	21.57	16.43	26.71	41.44	32.40	50.48
	H2	47.50	37.61	57.39	51.38	42.22	60.53	56.60	45.64	67.54

LF/HF	H1	1.46	0.97	1.96	1.17	0.53	1.81	1.09	0.41	1.77
	Tilt	4.85	3.62	6.08	4.44	2.92	5.96	1.83	1.11	2.56
	H2	1.60	0.90	2.29	1.29	0.58	2.00	1.04	0.49	1.60

H1 = first horizontal position, Tilt = 50-degree head-up position, H2 = second (recovery) horizontal position. LF_nu _= normalized low frequency, HF_nu _= normalized high frequency, LF/HF = low frequency to high frequency ratio. The protocol was completed three times, Control, Sham, and OMT.

**Figure 2:**
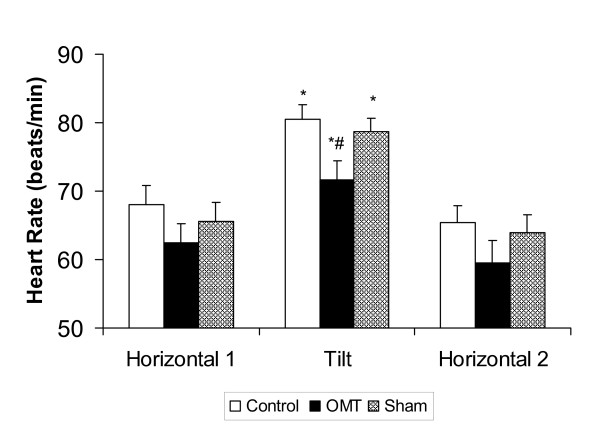
**Effect of body position and treatment on heart rate**. Values are mean ± SEM. * indicates a significant difference (p < 0.001) within a condition between H1 and Tilt positions; # indicates a significant difference (p < 0.001) between control and OMT and sham and OMT at Tilt position.

Figure 2 shows the effect of body position and treatment on heart rate. Passively changing body position from horizontal to the 50-degree head-up position resulted in a significant increase in heart rate for the control, sham, and OMT conditions, F(2,15) = 94.82, p < 0.001. Although heart rate tended to be lower in the OMT condition, the change in heart rate that occurred when the subjects assumed the head-up position was similar in all three conditions. Heart rate for all conditions returned to baseline levels when the subjects returned to the horizontal position.

**Figure 3:**
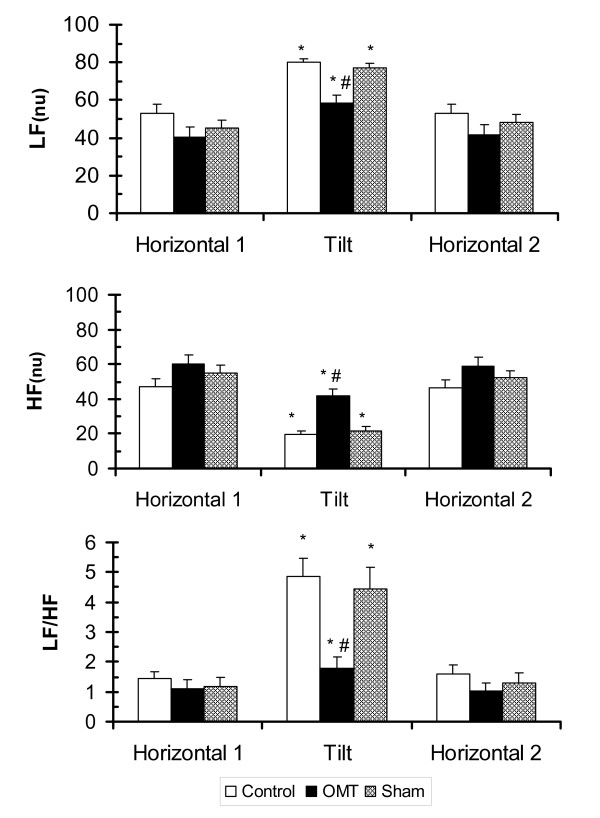
**Effect of body position and treatment on LFnu, HFnu and LF/HF ratio**. Values are mean ± SEM. LF = Low Frequency, HF = High Frequency, nu = normalized. * indicates a significant difference (p < 0.001) within a condition between H1 and Tilt positions; # indicates a significant difference (p < 0.001) between control and OMT conditions and sham and OMT conditions in the Tilt position.

The effects of body position and treatment on heart rate variability in the frequency domains are shown in Figure 3. Normalized power in the low frequency range (Figure 3 upper panel) significantly increased in the control and sham conditions when subjects were moved from horizontal to head-up tilt, F(2,15) = 47.35, p < 0.001. The magnitude of change was similar in the control and sham conditions. However, when OMT treatment was applied in the head-up position, the increase in LF_nu _was significantly less when compared to the increases in LF_nu _that occurrerd in the control and sham conditions (p < 0.001). LF_nu _returned to baseline in all three experimental conditions when the subjects were returned to the horizontal position.

In the head-up tilt position, normalized high frequency power (Figure 3, middle panel) in the control and sham conditions significantly decreased, F(2,14) = 43.005, p < 0.001. The change in HF_nu _was not as great in the OMT treatment condition (p < 0.001).

As shown in Figure 3 (lower panel), head-up tilt significantly increased the LF/HF ratio in all three conditions, F(2,15) = 30.49, p < 0.001, reflecting an increase in sympathetic tone. The increase in LF/HF ratio was due to an increase in LF_nu _and a decrease in HF_nu _power. Cervical myofascial release resulted in a lower LF/HF ratio in the head-up tilt phase by decreasing LF_nu _and increasing HF_nu _power in comparison to control and sham. The initiation of OMT in head-up tilt resulted in a change back, nearly to baseline, to a more parasympathetic tone. The LF/HF ratio returned toward baseline conditions when the subjects were returned to the horizontal position.

The LF/HF ratios in the head-up tilt position for the control condition and the sham condition were over 2.5 times the LF/HF ratio in the OMT condition, as can be seen in Table 1. This is a statistically significant difference (p < 0.001) between the control and sham conditions at tilt and the OMT condition at tilt.

**Figure 4:**
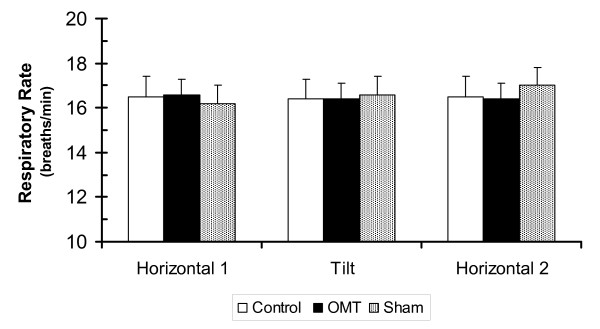
**Effect of body position and treatment on respiratory rate**. Values are mean ± SEM. There were no significant differences within or between conditions.

Heart rate variability and spectral indices of heart rate variability can be influenced by respiratory rate. Respiration was measured continuously throughout the study. As shown in Figure 4, respiration did not change with body position and was similar in all three conditions, F(2,15) = 0.65, p > 0.50.

## Discussion

Although the osteopathic profession has long recognized a relationship between the autonomic nervous system and the function of the body in health and disease, the ability to demonstrate a causal relationship between OMT and the autonomic nervous system has been problematic. Understanding the ability of manipulative medicine to influence sympathovagal balance and recognizing its limitations is critical in defining and refining OMT outcomes. The goal of the present study was to address this problem by using noninvasive measures of sympathovagal balance. This study tested the hypothesis that cervical myofascial release increased vagal tone. Using an experimental protocol in which a tilt table was used to set a range for sympathetic and vagal modulation of heart rate, the results of the study showed that the initiation of OMT in a head-up tilt position resulted in a change back, nearly to baseline levels, to a more parasympathetic tone. This represented a shift from a sympathetic to a parasympathetic environment, demonstrating the effect of the OMT treatment in overcoming the sympathetic tone. The results established a clear association between the effects of OMT and changes in autonomic activity.

Results of this study have demonstrated, for the first time, a quantitative relationship between osteopathic manipulation and sympathovagal balance. That is, cervical myofascial release shifts sympathovagal balance from the sympathetic to the parasympathetic nervous system. The present study simplifies the analysis and reduces nonspecific influences by putting the results of HRV in the context of changes that occur during passive head-up tilt. In this study, passive tilt was used to create a graded change in sympathovagal balance and it did this with minimal changes in central cardiovascular drive, muscle afferents nerve activity, respiration, and cortical activity.[13] The effect in this study of overcoming a maximally stimulated sympathetic tone created by the tilt position means that the observed parasympathetic response could only have come from the effect of the OMT. In effect, the OMT results are compared against changes in spectral HRV measures that occur in response to a physiological change in body position.

The mechanism for the effect of cervical myofascial release on autonomic balance was not investigated in this study. However, the role of neck afferents with projections to vestibular nuclei may be a possibility. The role of neck afferents and vestibulo-sympathetic reflexes in cardiovascular control is receiving increasing attention. It is well documented that head down neck flexion can activate a variety of autonomic reflexes that affect vascular control. In this regard, Lee et al[14] studied the effect of head down neck flexion on heart rate variability and found that this maneuver significantly affected autonomic balance as measured by the normalized LF/HF ratio.

Spectral measure of HRV and autonomic balance are subject to some variability due to their sensitivity to changes in hemodynamic state, stress, and health. It is generally recognized that respiration has an important effect on heart rate variability.[15] Respiration was continuously measured in this study and was not affected by body position or condition (control, treatment, sham). Therefore, the results of the study were not influenced by alterations in respiration.

The changes observed in heart rate and spectral analysis in response to passive head-up tilt in this study are comparable to the change observed in other studies.[10,16-18] Montano et al[16] used power spectrum analysis to assess the changes in sympathovagal balance during graded tilt. They found graded changes in normalized units of low and high frequency power and the LF/HF ratio. The magnitude of changes in their study (60-degrees head-up tilt) was very similar to the changes seen in our control subjects. Based on Figure 3 from Montano et al,[16] LF_nu _increased from approximately 40 in the horizontal position to 75 in the 60-degree head-up position. Normalized HF power decreased from approximately 50 to 20 and the LF/HF ratio increased from around 1 to 9 when the subjects were tilted. Inspection of Table 1 in the present study shows comparable changes for our control condition: LF_nu _increased from 53.2 in the horizontal position to 80.2 in the 50-degree head-up position. Normalized HF power decreased from 47.9 to 20.2 and the LF/HF ratio increased from 1.5 to 4.9 when the subjects were tilted. The difference between the two studies is the intervention of OMT in the present study. Further inspection of Table 1 in the present study shows that, when cervical myofascial release was applied in the tilt position, as much as 79% of the increase in the LF/HF ratio was abolished. This convincingly shows an effect of OMT on sympathovagal balance.

This study shows the utility of using HRV as a method for measuring autonomic change. It also demonstrates the positive association between OMT and the autonomic nervous system as the HRV changes with the initiation of OMT. This is the first time the association between OMT and autonomic activity has been quantified in this way, and establishes a plausible mechanism for how OMT works in the body. The change in HRV seen with initiation of OMT has physiological significance since it is being compared to pre-calibrated positional changes. In this study, OMT intervention is the only explanation for producing a significant vagal response in an otherwise strong sympathetic environment. The implications of this study are important in laying a foundation for understanding how OMT facilitates physiologic change, taking the use of OMT out of the arena of empiricism. The ability to use HRV as a surrogate for autonomic activity also highlights its use in sham controls where the concern has always been that the positioning of a subject or laying on of hands could somehow exert its own effect. In this study we observed that the sham and control conditions were not significantly different from each other.

## Conclusion

This study demonstrated quantitatively that cervical myofascial release shifts sympathovagal balance from the sympathetic to the parsympathetic nervous system. This study employed one particular type of OMT technique, cervical myofascial release. Future studies should be aimed at demonstrating the same effect on the autonomic nervous system using other techniques, such as high velocity/low amplitude. The subjects used in the present study were free of pathology. Studies showing the effect on autonomic change when OMT is applied in a disease state are needed for further understanding of the physiological mechanism.

## List of Abbreviations

OMT: Osteopathic Manipulative Treatment; HRV: Heart rate variability; ECG: electrocardiogram; LF: Low frequency; HF: High frequency; LF_nu_: Low frequency, normalized; HF_nu_: High frequency, normalized; LF/HF: Low frequency to high frequency ratio; IRB: Institutional review board; ANOVA: Analysis of variance.

## Competing interests

The authors declare that they have no competing interests.

## Authors' contributions

CEH originated and designed the study, performed the power analysis, conducted the study and performed OMT on subjects, contributed critical insights in interpretation of data, and had primary responsibility for writing the paper. CEH and BAB were coordinating investigators. BAB wrote the IRB proposal and also originated and designed the study, conducted the study and collected ECG data, cleaned and analyzed the data, contributed critical insights in interpretation of data, and participated in manuscript preparation. DI consulted on statistical analysis of the data and interpretation of outcomes as well as final review of the manuscript. MM performed OMT, consulted on the technique used in the study, and provided manuscript review. FKW contributed to study design, data entry, multivariate analysis of the data and interpretation of outcomes, and manuscript design including figures and manuscript review. All authors have read and approved the final version of the manuscript.

## References

[B1] Anderson RE, Seniscal C (2006). A comparison of selected Osteopathic treatment and relaxation for tension-type headaches. Headache.

[B2] Burns DK, Wells MR (2006). Gross range of motion in the cervical spine: The effects of Osteopathic muscle energy technique in asymptomatic subjects. JAOA.

[B3] Celander E, Koenig AJ, Celander DR (1968). Effect of Osteopathic manipulative therapy on autonomic tone as evidenced by blood pressure changes and activity of the fibrinolytic system. JAOA.

[B4] Sergueef N, Nelson KE, Glonek T (2002). The effect of cranial manipulation on the Traube-Hering-Mayer Oscillation as measured by Laser-Doppler Flowmetry. Altern Ther Health Med.

[B5] Kautzner J, Camm AJ (1997). Clinical relevance of heart rate variability. Clin Cardiol.

[B6] Sztajzel J (2004). Heart rate variability: a noninvasive electrocardiographic method to measure the autonomic nervous system. Swiss Med Wkly.

[B7] Electrophysiology TFESCNASP (1996). Heart rate variability. Standards of measurement, physiological interpretation, and clinical use. Eur Heart J.

[B8] Nakamura Y, Yamamoto Y, Muraoka I (1993). Autonomic control of heart rate during physical exercise and fractal dimension of heart rate variability. J Appl Physiol.

[B9] Pagani M, Lombardi F, Guzzetti S, Rimoldi O, Furlan R, Pizzinelli P, Sandrone G, Malfatto G, Dell'Orto S, Piccaluga E, Turiel M, Baselli G, Cerutti S, Malliani A (1986). Power spectral analysis of heart rate and arterial pressure variabilities as a marker of sympatho-vagal interaction in man and conscious dog. Circ Res.

[B10] Malliani A, Pagani M, Lombardi F, Cerutti S (1991). Cardiovascular neural regulation explored in the frequency domain. Circulation.

[B11] Chobanian AV, Bakris GL, Black HR, Cushman WC, Green LA, Izzo Jr JL, Jones DW, Materson BJ, Oparil S, Wright Jr JT, Roccella EJ (2003). National High Blood Pressure Education Program Coordinating Committee: The Seventh Report of the Joint National Committee on Prevention, Detection, Evaluation, and Treatment of High Blood Pressure.. Journal of the American Medical Association.

[B12] Graham KE (2001). Osteopathic Clinical Evaluation and Treatment of the Ten Most Common Musculoskeletal Presentations.

[B13] Cooke WH, Hoag JB, Crossman AA, Kuusela TA, Tahvanainen KU, Eckberg DL (1999). Human responses to upright tilt: a window on central autonomic integration. J Physiol (Lond).

[B14] Lee CM, Wood RH, Welsch MA (2001). Influence of head-down and lateral decubitus neck flexion on heart rate variability. J Appl Physiol.

[B15] Hayano J, Mukai S, Sakakibara M, Okada A, Takata K, Fujinami T (1994). Effects of respiratory interval on vagal modulation of heart rate. Am J Physiol.

[B16] Montano N, Ruscone TG, Porta A, Lombardi F, Pagani M, Malliani A (1994). Power spectrum analysis of heart rate variability to assess the changes in sympathovagal balance during graded orthostatic tilt. Circulation.

[B17] Furlan R, Piazza S, Dell'Orto S, Gentile E, Cerutti S, Pagani M, Malliani A (1993). Early and late effects of exercise and athletic training on neural mechanisms controlling heart rate. Cardiovasc Res.

[B18] Martinelli FS, Chacon-Mikahil MPT, Martins LEB, Lima-Filho EC, Golfetti R, Paschoal MA, Gallo-Junior L (2005). Heart rate variability in athletes and nonathletes at rest and during head-up tilt. Braz J Med Biol Res.

